# *Lactobacillus Fermentum* ZS40 prevents secondary osteoporosis in Wistar Rat

**DOI:** 10.1002/fsn3.1824

**Published:** 2020-08-13

**Authors:** Xinhong Liu, Jian‐Bo Fan, Jing Hu, Fang Li, Ruokun Yi, Fang Tan, Xin Zhao

**Affiliations:** ^1^ Chongqing Collaborative Innovation Center for Functional Food Chongqing University of Education Chongqing China; ^2^ Chongqing Engineering Research Center of Functional Food Chongqing University of Education Chongqing China; ^3^ Chongqing Engineering Laboratory for Research Development of Functional Food Chongqing University of Education Chongqing China; ^4^ College of Biological and Chemical Engineering Chongqing University of Education Chongqing China; ^5^ Department of Orthopedics Chengdu Qingbaijiang District Traditional Chinese Medicine Hospital Chengdu China; ^6^ Department of Public Health Our Lady of Fatima University Valenzuela City Philippines

**Keywords:** bone biology, *Lactobacillus fermentum* ZS40, rat, secondary osteoporosis

## Abstract

Using retinoic acid to inducer, we successfully established a rat model of secondary osteoporosis and verified the preventive effect of *Lactobacillus fermentum* ZS40 (ZS40) on secondary osteoporosis. Serum biochemical indicators showed that ZS40 can effectively slow down bone resorption caused by retinoic acid, increase blood content of calcium, phosphorus, bone alkaline phosphatase, bone gla protein, and insulin‐like growth factor 1, and decrease blood content of tartrate‐resistant acid phosphatase (TRAP) 5b. qRT‐PCR results showed that ZS40 could upregulate mRNA expressions of *β‐catenin*, *Wnt10b*, *Lrp5*, *Lrp6*, *Runx2*, *ALP*, *RANKL*, and *OPG*, and downregulate mRNA expression of *DKK1*, *RANK*, *TRACP,* and *CTSK* in the rats’ spinal cord. Results following TRAP staining showed that ZS40 could slow down retinoic acid‐induced formation of osteoclasts. Micro‐CT results showed that ZS40 could reduce Tb.Sp, increase BV/TV, Tb.N, Tb.Th, and ultimately increase bone mineral density of rats in vivo. These findings indicate that ZS40 might have a potential role in preventing retinoic acid‐induced secondary osteoporosis in vivo.

## Graphical abstract

Lactobacillus fermentans zs40 is a newly discovered microorganism, which can prevent secondary osteoporosis in Wistar rats and has good probiotic potential.

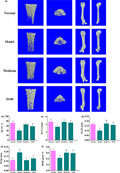

## 1 INTRODUCTION

Osteoporosis (OP) is a common complex metabolic disease, characterized by reduced bone mass, degeneration of bone tissue microstructure, and increased bone fragility, which, in light of demographic changes worldwide, is becoming an increasing burden on global healthcare services (Aspray & Hill, 2019; Gennari, Rotatori, Bianciardi, Nuti, & Merlotti, 2016). According to the pathogenesis and causes, OP can be divided into two major types, namely primary and secondary OP. In addition to idiopathic causes, primary OP is further divided into type I and type II. Type I is a high conversion type, arising primarily from estrogen deficiency, and thus also known as postmenopausal OP. Type II is a low conversion type, mainly caused by advancing age, and thus also known as senile OP (Glaser & Kaplan, 1997). Secondary OP is an adverse reaction, secondary to long‐term medication for treatment of diseases such as hyperthyroidism, diabetes, and malignant tumors (Emkey & Epstein, 2014; Hudec & Camacho, 2013; Stein & Shane, 2003).

Glucocorticoids are widely used in chronic noninfectious inflammatory diseases, allergies, and following organ transplantation. One of the most serious side effects of glucocorticoid treatment is OP (Cooper er al., 2016; Krela‐Kaźmierczak, Szymczak, Lykowska‐Szuber, Eder, & Linke, 2016; Whittier & Saag, 2016). Even physiological doses of glucocorticoids can cause bone loss. Glucocorticoid‐induced OP is the most common form of secondary OP (Canalis, Mazziotti, Giustina, & Bilezikian, 2007; Hardy, Zhou, Seibel, & Cooper, 2018). Use of other immunosuppressants following organ transplantation can also lead to secondary OP (Dounousi, Leivaditis, Eleftheriadis, & Liakopoulos, 2015; Faraj et al., 2016; Kulak, Cochenski‐Borba, Kulak, & Ribeiro‐Custódio, 2012). One of the side effect of retinoic acid, a drug used to treat malignant tumors, is induction of secondary OP (Broulík, Raška, & Brouliková, 2013; Hotchkiss, Latendresse, & Ferguson, 2006; Oršolić et al., 2014; Yang et al., 2016; Zhao et al., 2016).

Treatment and care for OP require enormous human and material resources, resulting in heavy burden on affected families, and society in general. Commercially available anti‐OP drugs, such as bisphosphonate and parathyroid hormone, are costly and have their own side effects, including creation of strong dependency and inability to fully restore bone metabolism balance (Wang et al., 2019). Some studies have shown that the intestinal colonization of probiotics in mice can affect bone formation and remodeling, and has a certain preventive effect against OP (Amin, Boccardi, Taghizadeh, & Jafarnejad, 2019; Collins, Rios‐Arce, Schepper, Parameswaran, & McCabe, 2017). Related experiments have shown that the probiotic *L. rhamnosus* can increase the expression level of butyrate and Wnt10b in mice, thereby positively regulates bone formation (Tyagi et al., 2018).

*Lactobacillus fermentum* ZS40 (ZS40) is a lactic acid bacterium isolated and identified by our research team in naturally fermented yak yogurt. In this study, we used retinoic acid to establish a rat model of secondary OP, and investigate the preventive effect of ZS40 on OP. We found that ZS40 supplementation increased the expression of osteogenic marker genes and stimulated bone formation. The results might provide a new and efficient preventive treatment strategy for OP.

## 2 MATERIALS AND METHODS

### 2.1 Laboratory strain

*Lactobacillus fermentum* ZS40 is preserved in the China General Microbiological Culture Collection Center (CGMCC No. 18226), Beijing, China. It was isolated from yak yogurt in Xinjiang province, China.

### 2.2 Rat in vivo model of osteoporosis

Forty‐eight‐week‐old female Wistar rats (Laboratory Animal Center of Chongqing Medical University, Chongqing, China) of similar body weight (250‐300 g) were randomly divided into four groups of 10 rats each—normal, model, medicine, and ZS40. The rats were acclimated to laboratory conditions for 1 week before initiating the prophylactic treatment. Rats in the normal and model groups were daily administered intragastrically with 1 ml of saline per 100 g body weight for 2 weeks. Rat in the medicine and ZS40 groups were administered daily intragastrically 1 ml with 10^10^ CFU/kg of ZS40 per 100 g body weight for 2 weeks. All groups except the normal group also received retinoic acid (80 mg/kg/d) intragastrically from day 14 for 4 weeks. The control (normal) group continued to receive an equal volume of saline. During these 4 weeks, the ZS40 group continued to receive daily the original dose of bacteria, while rats in the medicine group were injected via the tail vein with one dose of 0.5 ml per 100 g body weight of zoledronic acid (Chiatai Tianqing). After 4 weeks, all rats were fasted for 24 hr and were then sacrificed by cervical dislocation. Serum and the spinal cord collected from the rats were stored at − 80°C, pending further analyses. Tibia and femur were collected and fixed in 10% (v/v) buffered formaldehyde for histological observations and micro‐computed tomography (micro‐CT). The protocol for these experiments was approved by the Ethics Committee of Chongqing Collaborative Innovation Center for Functional Food (201904027B), Chongqing, China.

### 2.3 Determination of serum calcium and phosphorus biochemical indicators

The collected rats' blood was centrifuged at 4,000 rpm for 10 min, and the supernatant (serum) was collected. Serum levels of calcium and phosphorus were determined following the kit's instructions (Nanjing Jiancheng Bioengineering Institute, Nanjing, Jiangsu, China).

### 2.4 Determination of serum cytokines BAP, BGP, IGF‐1R, TRACP‐5b, and GABA

Cytokines levels were assayed using BAP (ml037086), BGP (ml002883), IGF‐1R (ml059459), TRACP‐5b (ml003177), and GABA (ml064273) cytokine assay kits (Shanghai Enzyme‐linked Biotechnology Co., Ltd.).

### 2.5 Quantitative real‐time PCR (qRT‐PCR) Assay

Total RNA was isolated from the spinal cords by TRIzol reagent (Invitrogen). One microliter of oligo(dt)18 primer (500 ng) and 1.0 μl of total RNA (1.0 μg) was added to 10.0 μl of nuclease‐free water and heated on a gradient PCR instrument for 5 min at 65°C, according to the manufacturer's recommendations (RevertAid First‐Strand cDNA Synthesis Kit; Thermo Fisher Scientific, Inc.). 2 μl of cDNA template was mixed with 10 μl of SYBR Green PCR Master Mix (Thermo Fisher Scientific) and 1 μl each of upstream and downstream primers. The system was reacted at 95°C for 60 s, and then through 40 cycles of 95°C for 30 s, Annealing Temperature for 30 s, 72°C for 30 s. Finally, the DNA was detected at 95°C for 30 s and 5°C for 35 s. The 2^‐ΔΔCt^ method was used to determine the level of relative gene expression. Table 1 lists the sequences of the PCR primer used.

**TABLE 1:** Sequences of primers used in this study

Gene name	Sequence
*β‐actin*	Forward: 5′‐ TCAGGTCATCACTATCGGCAAT −3′
	Reverse: 5′‐ AAAGAAAGGGTGTAAAACGCA −3′
*β‐catenin*	Forward: 5′‐ GGTGAAAATGCTTGGGTCGC −3′
	Reverse: 5′‐ AGATCTGAAGGCAGTCTGTCGTAA −3′
*Wnt10b*	Forward: 5′‐ GTGGGAATGGGGTGGCTGTA −3′
	Reverse: 5′‐ CCGCATTCTCGCCTGGAT −3′
*Lrp5*	Forward: 5′‐ GCATCATCCTGTCCCTCTTCG −3′
	Reverse: 5′‐ GACCGTGCTGTGAGCCACC −3′
*Lrp6*	Forward: 5′‐ ACAGACTGGAGCCGACGCA −3′
	Reverse: 5′‐ GCCAAGCAAAGGTGGGAGC −3′
*Runx2*	Forward: 5′‐ GAACCAAGAAGGCACAGACAGAA −3′
	Reverse: 5′‐GGCGGGACACCTACTCTCATACT −3′
*ALP*	Forward: 5'‐ GCGACAGCAAGCCCAAGAG −3'
	Reverse: 5'‐ CTCCAGCCGTGTCTCCTCG −3'
*RANKL*	Forward: 5'‐ TGGAGAGCGAAGACACAGAAGC −3'
	Reverse: 5'‐ GGTGAGGTGAGCAAACGGC −3'
*OPG*	Forward: 5′‐ AATTGGCTGAGTGTTCTGGTGG −3′
	Reverse: 5′‐ GCTGGAAAGTTTGCTCTTGCG −3′
*DKK1*	Forward: 5′‐ GGCTCTGTCTGCCTCCGATC −3′
	Reverse: 5′‐ GCCTTTCCTCCTGTGCTTGG −3′
*RANK*	Forward: 5′‐ GGCTTCTTCTCAGATGTCTTTTCG −3′
	Reverse: 5′‐ TGATTCCGTCGTCCCTTGGT −3′
*TRACP*	Forward: 5′‐ GTGGCTGTGGGTGACTGGG −3′
	Reverse: 5′‐ CAAAGGTCTCCTGGAACCTCTTG −3′
*CTSK*	Forward: 5′‐ AAGGCAGCTAAGTGCAGAGGG −3′
	Reverse: 5′‐ GGTTCACATTATCACGGTCGC −3′

### 2.6 Histological observations

The tibia and femur were removed immediately after sacrificing the rats and fixed in 10% (v/v) buffered formaldehyde. The proximal tibia femur was embedded in paraffin, sectioned, stained with tartrate‐resistant acid phosphatase (TRAP), and observed on a BX43 microscope (Olympus). The number of osteoclasts with three or more nuclei was counted in six randomly selected fields, and the average was calculated to represent the number of osteoclasts in each histological section (Zeng et al., 2017).

### 2.7 Bone Imaging by micro‐CT

To image the entire tibia and femur, Bruker MicroCT Skyscan 1272 system was used with isotropic voxel size of 10.0 μm. Scans were conducted in 4% paraformaldehyde and used an X‐ray tube potential of 60 kV, intensity of 166 μA, and exposure time of 1,700 ms. For trabecular bone analysis of the distal tibia and femur, the upper 3 mm region, beginning 0.8 mm proximal to the most proximal central epiphysis of the femur, was contoured (Longo, Salmon, & Ward, 2017).

### 2.8 Statistical analysis

Data are expressed as mean ± *SD*. The SPSS software version 22 (IBM Corporation) was used for analysis of variance (ANOVA) with the post hoc Duncan's new multiple‐range test. Differences at *p* < .05 were considered statistically significant. All figures were drawn using Origin 8.0 software (Li et al., 2017).

## 3 RESULTS AND DISCUSSION

### 3.1 Serum levels of calcium and phosphorus biochemical indicators

Table 2 presents the levels of calcium and phosphorus in the serum of the rats, showing that levels in the model group were the lowest, while those in the medicine and ZS40 groups were significantly higher when compared with the control and model groups.

**TABLE 2:** Levels of calcium and phosphorus in rats' serum

Group	Normal	Model	Medicine	ZS40
Calcium (mmol/L)	1.26 ± 0.10 ^a^	1.10 ± 0.15 ^a^	1.15 ± 0.14 ^a^	1.78 ± 0.24 ^b^
Phosphorus (mmol/L)	1.94 ± 0.23 ^a^	1.72 ± 0.16 ^a^	2.23 ± 0.32 ^b^	2.40 ± 0.30 ^b^

Note

Values presented are the mean ± *SD* (*n* = 10/group). Mean values with different superscript letters in the same row are significantly different (*p* < .05). Normal: normal rats control; Model: rats treated with retinoic acid (80 mg/kg/d); Medicine: rats treated with retinoic acid (80 mg/kg/d) and single treatment with 0.5 ml/100 g zoledronic acid; ZS40: rats treated with retinoic acid (80 mg/kg/d) and 1 ml/100 g with 10^10^ CFU/kg of *L. fermentum* ZS40.

### 3.2 Serum levels of cytokines BAP, BGP, IGF‐1R, TRACP‐5b, and GABA

Serum cytokine detection assays showed that serum levels of BAP, BGP, IGF‐1R, and GABA in model rats were the lowest, while these were significantly higher in the medicine and ZS40 groups, when compared with the normal (control) and model groups. On the other hand, serum level of TRACP‐5b in the model group was the highest while it was significantly lower in the medicine and ZS40 groups when compared with the normal and model groups (Table 3).

**TABLE 3:** Levels of BAP, BGP, IGF‐1R, TRACP‐5b, and GABA serum of rats

Group	Normal	Model	Medicine	ZS40
BAP (ng/ml)	12.22 ± 0.99 ^b^	10.61 ± 1.02^a^	13.89 ± 1.55^c^	14.63 ± 1.73 ^c^
BGP (ng/ml)	2.99 ± 0.49 ^b^	1.53 ± 0.47^a^	4.02 ± 1.15^c^	4.33 ± 1.43^c^
IGF−1R (pg/ml)	690 ± 199.72 ^b^	262.86 ± 81.72^a^	1,036.43 ± 91.96^c^	1,052.86 ± 96.32 ^c^
TRACP−5b (ng/ml)	0.74 ± 0.39 ^a^	2.24 ± 0.67^b^	0.40 ± 0.20^a^	0.78 ± 0.43^a^
GABA (umol/l)	7.52 ± 0.18 ^b^	7.08 ± 0.31^a^	7.72 ± 0.27^bc^	7.88 ± 0.39^c^

Note

Values presented are the mean ± *SD* (*n* = 10/group). Mean values with different superscript letters in the same row are significantly different (*p* < .05). Normal: normal rats control; Model: rats treated with retinoic acid (80 mg/kg/d); Medicine: rats treated with retinoic acid (80 mg/kg/d) and a single dose of 0.5 ml/100 g zoledronic acid; ZS40: rats treated with retinoic acid (80 mg/kg/d) and 1 ml/100 g with 10^10^ CFU/kg of *L. fermentum* ZS40. BAP: bone‐specific alkaline phosphatase. BGP: osteocalcin. IGF‐1R: insulin‐like growth factor‐1 receptor. TRACP‐5b: tartrate‐resistant acid phosphatase 5b. GABA: γ‐ aminobutyric acid.

### 3.3 Expression of RNA in the spinal cord

**FIGURE 1:**
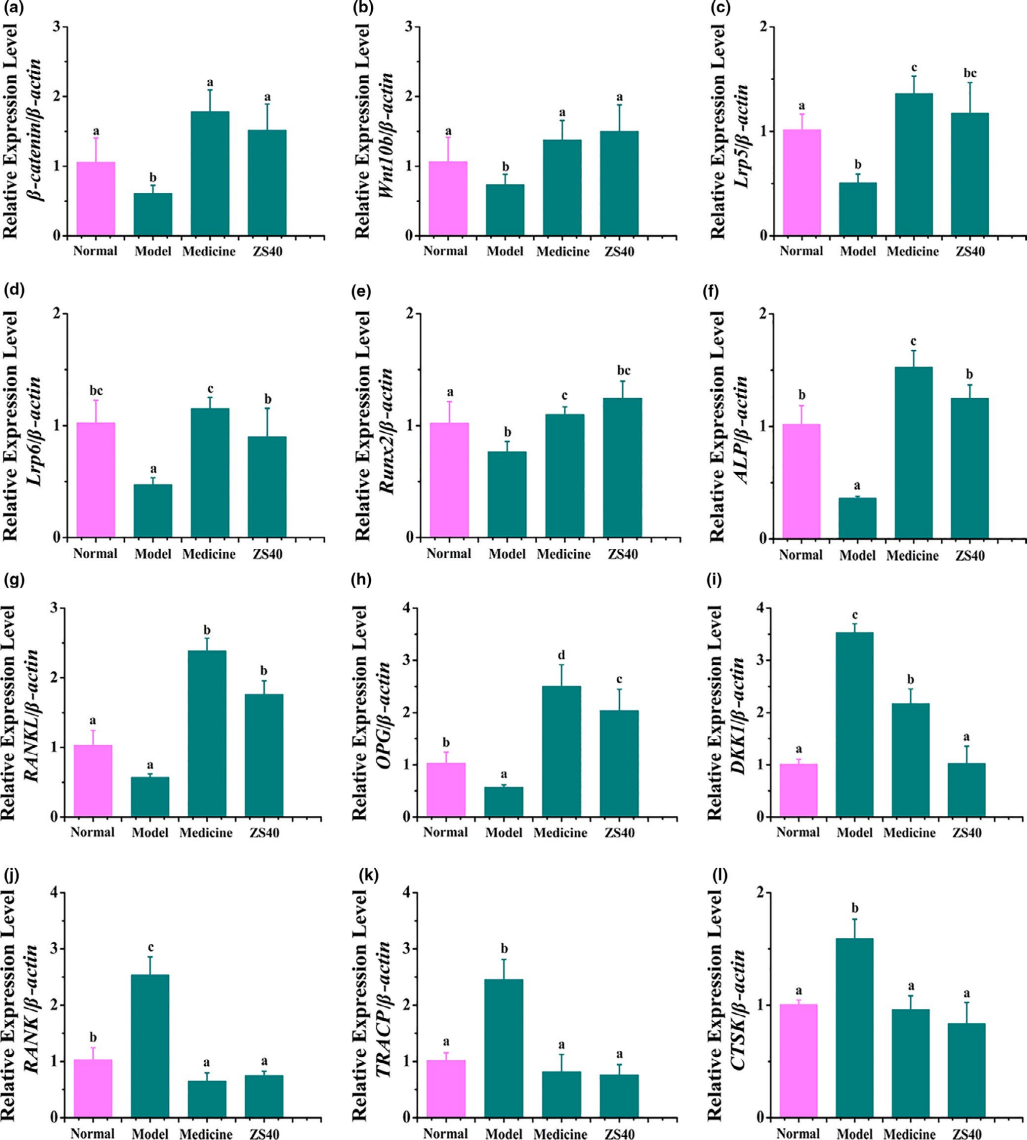
*β‐catenin*, *Wnt10b*, *Lrp5*, *Lrp6*, *Runx2*, *ALP*, *RANKL*, *OPG*, *DKK1*, *RANK*, *TRACP*, and *CTSK* mRNA expression in the spinal cord of rats. Different letters over the bars within each panel indicate significant difference between the groups (*p* < .05). (a) β‐catenin, a Wnt signaling pathway key protein. (b) Wnt10b, Wnt signaling pathway key protein. (c) Lrp5, low‐density lipoprotein receptor‐related protein 5. (d) Lrp6, low‐density lipoprotein receptor‐related protein 6. (e) Runx2, runt‐related transcription factor 2. (f) ALP, alkaline phosphatase. (g) RANKL, receptor activator of nuclear factor‐κB ligand. (h) OPG, osteoprotectin. (i) DKK1, dickkopf‐related protein 1. (j) RANK, receptor activator of nuclear factor‐κB. (k) TRACP, tartrate‐resistant acid phosphatase. (l) CTSK, cathepsin K

Expression levels of mRNA of *β‐catenin*, *Wnt10b*, *Lrp5*, *Lrp6*, *Runx2*, *ALP*, *RANKL,* and *OPG* were the lowest in spinal cords from model rats and highest in the spinal cords of the medicine and ZS40 groups. On the other hand, expression levels of *DKK1*, *RANK*, *TRACP,* and *CTSK* were the highest in the model group and the lowest in the medicine and ZS40 groups. The effect of ZS40 was strong, and the expression of bone marker genes in spinal cord of this group was close to those of the medicine and normal rats’ groups (Figure 1).

### 3.4 Pathological observation of rats' femur and tibia

**FIGURE 2:**
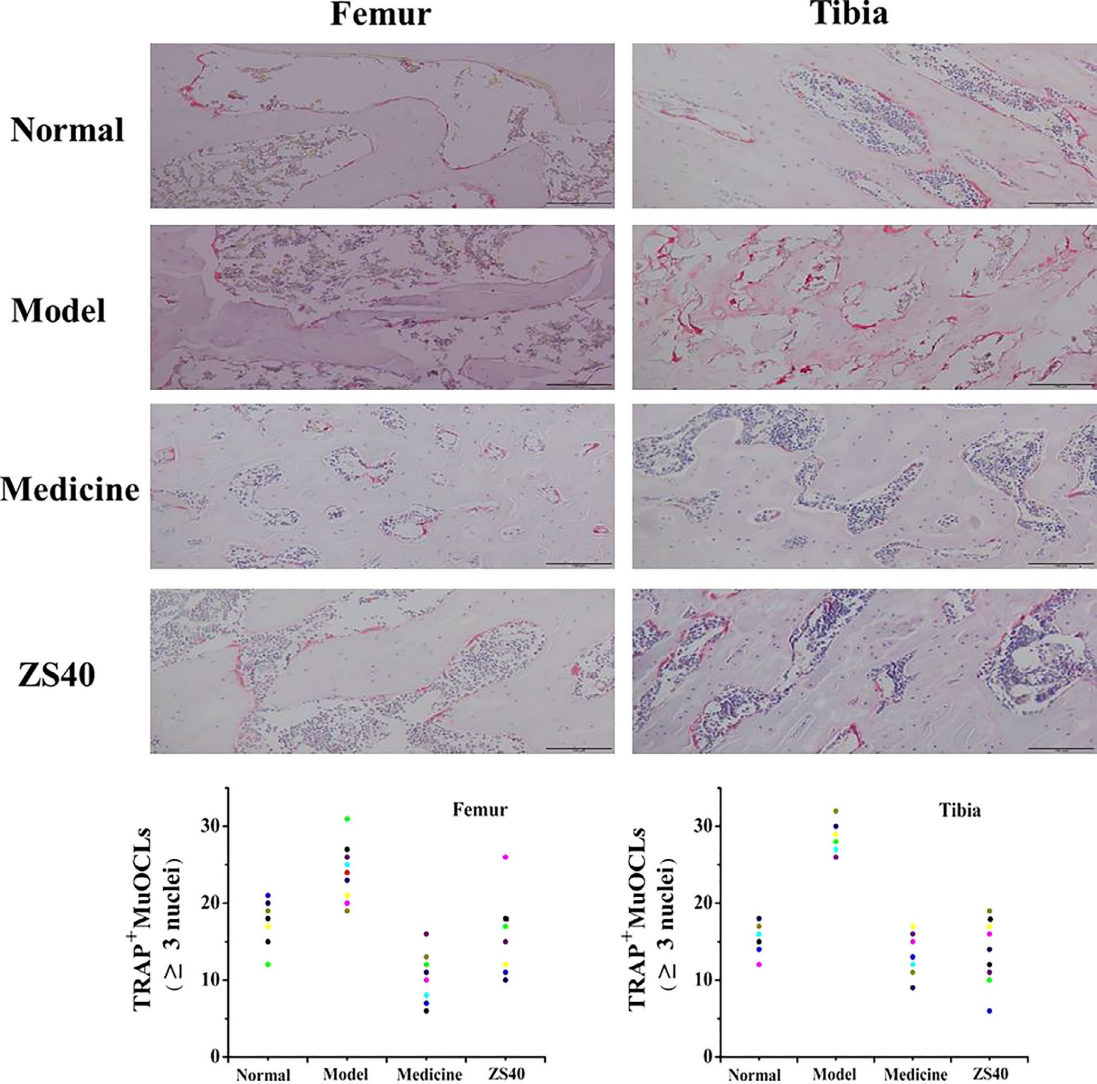
Pathological observation of rats' femur and tibia stained with TRAP. Magnification: 100×. Normal: normal rats control; Model: rats treated with retinoic acid (80 mg/kg/d); Medicine: rats treated with retinoic acid (80 mg/kg/d) and a single dose of 0.5 ml/100 g zoledronic acid; ZS40: rats treated with retinoic acid (80 mg/kg/d) and 1 ml/100 g with 10^10^ CFU/kg of *L. fermentum* ZS40

Figure 2 shows histological micrographs of the rats' femur and tibia. In the normal group, the femur and tibia showed normal number of osteoclasts, morphology, and degree of fusion. The number of osteoclasts in the model group rats increased significantly, and a large number of fused giant multinucleated cells appeared. The medicine and ZS40 groups, on the other hand, were similar to the normal group, and the number of osteoclasts was significantly reduced back to the level of the normal group.

### 3.5 Micro‐CT of rats' femur

**FIGURE 3:**
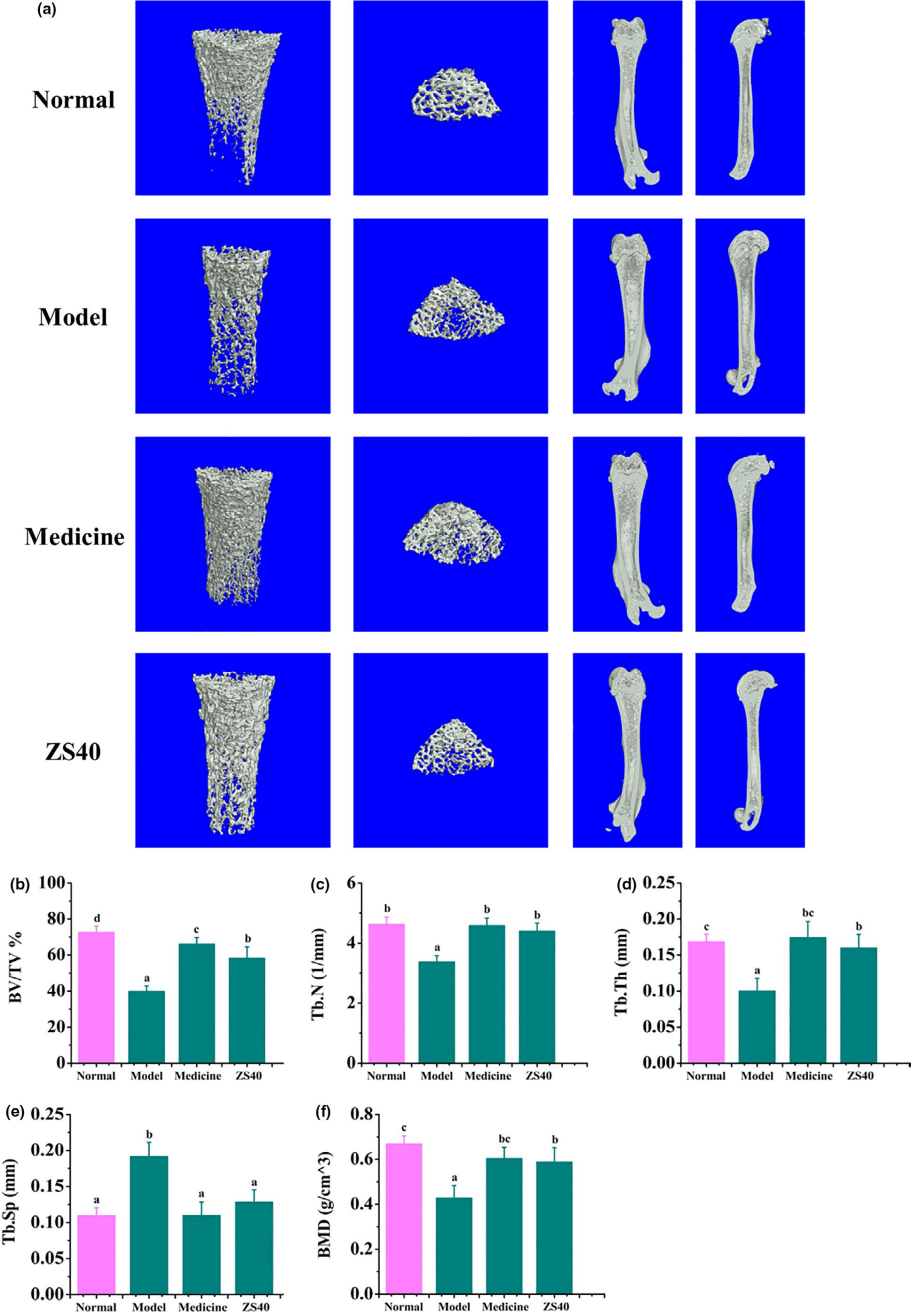
Micro‐CT results of rats' femur. Different letters over the bars within each panel indicate significant difference between the groups (*p* < .05). (a) All images are representative of the respective groups (*n* = 6). (b) Percent bone volume (BV/TV). (c) Trabecular number (Tb.N). (d) Trabecular thickness (Tb.Th). (e) Trabecular separation (Tb.Sp). (f) Bone mineral density (BMD)

Figure 3 shows the micro‐CT results for the rats' femurs. In the normal group, the femurs had normal values for percent bone volume (BV/TV), trabecular number (Tb.N), trabecular thickness (Tb.Th), trabecular separation (Tb.Sp), and bone mineral density (BMD). In the model group, values of BV/TV, Tb.N, Tb.Th, and BMD of the femur were the lowest and those of Tb.Sp were the highest. The femur of the medicine and ZS40 groups were similar to the normal group. Values of BV/TV, Tb.N, Tb.Th, and BMD have significantly increased and those of Tb.Sp have significantly decreased.

## 4 DISCUSSION

Retinoic acid is a derivative of vitamin A. It is a natural inhibitor of human tumors and is commonly used in the treatment of many diseases (Fischer‐Huchzermeyer et al., 2017; Rizzoli & Biver, 2018). However, a large body of research has shown that long‐term use of retinoic acid can cause a variety of diseases, with OP being the most important one (Conserva, Anelli, Zagaria, Specchia, & Albano, 2019; Petkovich, Heersche, Aubin, Grigoriadis, & Jones, 1987). We therefore chose to treat rats with retinoic acid to create a secondary OP model. Recently, various studies have been published in both health and pathological models on supplementing probiotics to enhance bone health, and it is common to treat probiotics in experimental animals 2 weeks before the start of the experiment to study the preventive effects of probiotics. Most of the studies involve the treatment of experimental animals by mixing a variety of probiotics or single probiotics with other reagents. However, we treated rat with ZS40 2 weeks before the experiment began, which provided a more direct explanation of the preventive effect of ZS40.

The related mechanism of the positive correlation between probiotics intake and osteoporosis amelioration is further investigated in vitro. Runx2 is a bone‐specific transcription factor that is a key factor in regulating osteoblast differentiation and promoting bone formation. As a marker of early osteogenesis, ALP is often used to detect osteogenesis. In this study, we found that ZS40 significantly increased mRNA levels of Runx2 and ALP, suggesting that ZS40 may promote osteogenic differentiation in rats. On the other hand, RANKL binding induces osteoclast differentiation and activation, the interaction between RANK and RANKL regulates genes related to osteoclast formation such as TRACP and CTSK. The results showed that ZS40 could downregulate the expression of RANK and inhibit the expression of osteoclast related genes TRACP and CTSK, suggesting that ZS40 may inhibit the differentiation of rat osteoclasts. Our study directly detected the expression of related genes in rats, which had certain physiological correlation.

The body's bone mass is in a constant dynamic balance between bone formation and bone resorption. Once this dynamic balance is broken, bone resorption surpasses bone formation, leading to the development of OP (Chen et al., 2017; Christen & Müller, 2017). During the loss of bone mass, mononuclear osteoclasts fuse to form giant multinucleated cells (Xing, Xiu, & Boyce, 2012). Tartrate‐resistant acid phosphatase (TRAP), being a specific marker enzyme for osteoclasts, can show their status when used. This study showed that ZS40 can reduce the number of multinucleated osteoclasts in tibia and femur, suggesting that ZS40 can inhibit the process of osteoclast differentiation in rat. In the treatment and ZS40 groups, the number of osteoclasts was similar to that in the normal group. This showed that ZS40 could prevent the development of OP caused by excessive retinoic acid drug use, and its effect was similar to that of zoledronic acid treatment in the medicine group. Similar results were also found in the determined of TRACP‐5b in rat serum, which further demonstrated the accuracy of our histological observations results.

Micro‐CT is a technique that combines noninvasive features of imaging and high‐resolution histological detection. Due to the relatively obvious difference between bones and other body tissues in X‐ray attenuation coefficients, micro‐CT is particularly suitable for bone imaging and can act as a direct indicator of bone mass (Buhmann, Becker, Duerr, Reiser, & Baur‐Melnyk, 2009; Jammal, Territoriale, Abate, & Missana, 2010). Previous work has indicated that the intestinal microbiota may impact bone health. Rodents and birds treated with prebiotics, such as inulin, oligofructose, and galactooligosaccharides, can also display increased bone density (Abrams et al., 2005; Coxam, 2007). For example, galactooligosaccharide intake resulted in a dose‐dependent increase in rat femur trabecular bone volume (Weaver et al., 2011). In our experiments, BV/TV, Tb.N, Tb.Th, and BMD of the femur were the lowest in the model group, and Tb.Sp was the highest. This clearly indicates that our model was successfully established. All these values returned to normal in the femurs of rats in the medicine and ZS40 groups, showing again that ZS40, which was isolated and purified in our laboratory, has good preventive effect against secondary OP, and the effect is similar to that of Zoledronic acid. Our work showed a positive regulatory effect of SZ40 on bone formation, but how it affects bone remodeling is unclear. Previous work has indicated that some probiotics could secrete molecules that reduce TNF‐α activity in vitro (Thomas et al., 2012). Such a factor could also cross the gut epithelium and affect local, systemic, and bone marrow immune responses and/or have direct effects on osteoclasts. Future studies will examine the specific components of ZS40 secretion and how they directly affect bone formation and absorption.

Probiotics are a single or a collection of well‐defined mix of active microorganisms that can be beneficial to the host when colonizing its body and changing the composition of its flora in the colonized body part. By regulating the host's mucosal and systemic immune functions or by adjusting the balance of the intestinal flora, probiotics promote the absorption of nutrients and maintain the health of the intestinal tract, thereby considered beneficial for the health (Shokryazdan, Jahromi, Liang, & Ho, 2017; Williams et al., 2010).

Our research team carried out preliminary experiments on ZS40, showing that after treatment with artificial gastric juice and bile salt, survival rates of ZS40 were 79.32% and 15.31%, respectively. We also used long‐term gastric irrigation to allow ZS40 colonize the intestinal tract of rats. Experimental results showed that ZS40 could indeed prevent the occurrence of secondary OP. Related experiments have shown that the probiotic *L. rhamnosus* could increase the expression levels of butyrate and Wnt10b in mice, thereby positively regulating bone formation. Using ELISA, we have also showed that ZS40 could increase the level of γ‐aminobutyric acid in rats’ serum (Table 3). However, whether ZS40 is directly involved in Wnt/ β‐catenin and OPG/ RANK/ RANKL signaling pathways and how it affects bone formation, remain unclear. This will be the focus of our subsequent research.

## 5 CONCLUSIONS

This study investigated the preventive effects of ZS40 on secondary OP in rats. We showed that the bacterium could effectively improve the expression of osteogenic marker genes in serum and spinal cord, and promote bone formation in rats in vivo. This study has built a foundation for further research on ZS40. Since only animal experiments were carried out in this study, future human clinical trials will be needed to confirm the preventive effects of ZS40 on secondary OP.

## CONFLICT OF INTEREST

The authors have declared that no competing interest exists.

## ETHICAL STATEMENT

The protocol for these experiments was approved by the Ethics Committee of Chongqing Collaborative Innovation Center for Functional Food (201901001B, Chongqing, China).
